# Manual therapy versus therapeutic exercise in non-specific chronic neck pain: a randomized controlled trial

**DOI:** 10.1186/s13063-020-04610-w

**Published:** 2020-07-28

**Authors:** Carlos Bernal-Utrera, Juan Jose Gonzalez-Gerez, Ernesto Anarte-Lazo, Cleofas Rodriguez-Blanco

**Affiliations:** 1 Doctoral Program in Health Sciences, University of Seville, Seville, Spain; 2 Fisiosur I+D Research Institute, Garrucha, Almería Spain; 3 Department Nursing, Physiotherapy and Medicine, University of Almeria, Almeria, Spain; 4 Centre of Precision Rehabilitation for Spinal Pain (CPR Spine), School of Sport, Exercise and Rehabilitation Sciences, University of Birmingham, Birmingham, UK; 5 Clinic San Vicente, Madrid, Spain; 6 Department of Physiotherapy, Faculty of Nursing, Physiotherapy and Podiatry, University of Seville, Seville, Spain

**Keywords:** Neck pain, Chronic pain, Exercise therapy, Musculoskeletal manipulations, Physical therapy specialty, Randomized controlled trial

## Abstract

**Background:**

Nonspecific chronic neck pain is a fairly common disorder that causes a great impact, and it is greatly influenced by psychosocial factors. Among a number of treatment modalities described for its management, the most common approach is based on manual therapy and specific therapeutic exercise, which have shown a moderate effect on subjects with chronic non-specific neck pain. However, the effect times of these treatments have not been accurately detailed. Our study aims to break down and compare the effects of two experimental treatments based on manual therapy and therapeutic exercise.

**Methods:**

The short-term and mid-term changes produced by different therapies on subjects with non-specific chronic neck pain were studied. The sample was randomized divided into three groups: manual therapy, therapeutic exercise, and placebo. As dependent variables of our research, we studied (a) pain, based on the visual analog scale and the pressure pain threshold, and (b) cervical disability, through the Neck Disability Index (NDI). Outcomes were registered on week 1, week 4, and week 12. The findings were analyzed statistically considering a 5% significance level (*P* ≤ 0.05).

**Results:**

No statistically significant differences (*P* 0.05) were obtained between the experimental groups, if they exist against the control group. Nonetheless, we found that manual therapy improved perceived pain before than therapeutic exercise, while therapeutic exercise reduced cervical disability before than manual therapy. Effect size (*R*^2^) shows medium and large effects for both experimental treatments.

**Conclusion:**

There are no differences between groups in short and medium terms. Manual therapy achieves a faster reduction in pain perception than therapeutic exercise. Therapeutic exercise reduces disability faster than manual therapy. Clinical improvement could potentially be influenced by central processes.

**Trial registration:**

Brazilian Clinical Trial Registry, RBR-2vj7sw. Registered on 28 November 2018.

## Background

Nonspecific neck pain is pain located in the lateral and posterior neck that does not show pathognomonic signs and symptoms [1]. When the duration of symptoms is greater than 12 weeks of evolution, it acquires the value of chronicity, being denominated non-specific chronic neck pain (NCNP) [2]. It is a common disorder, which generates a great impact and socio-economic cost [3, 4]. The number of prevalent cases of neck pain worldwide was estimated to be 288.7 million, and the number of years lived with disabilities due to neck pain in 2017 worldwide was estimated to be approximately 28.6 million [4].

Underlying mechanisms of NCNP maintenance, recurrence, and progression are not clear, but they could be associated with a deficit and alteration of the proprioception of the neck muscles that play a decisive role in the cervical joint position and motor control of the head [5].

In addition, strong relationships have been detected between neck pain and psychosocial factors such as catastrophism, stress, anxiety, and depression that influence the sensation of pain [6, 7].

Many studies have evaluated the effect of manual techniques and therapeutic exercise in patients with non-specific chronic neck pain, with the aim of checking its usefulness for treatment of this clinical condition [8, 9].

However, there is less evidence of differences between time of action and duration of its effects. Manual therapy involves neurophysiological mechanisms such as reduction in inflammatory biomarkers, decreased spinal excitability and pain sensitivity, modification of activity in cortical areas involved in pain processing, and excitation of sympathetic nervous system [10]. Instead, although therapeutic exercise has also shown neurophysiological effects, it involves reorganization in motor patterns, structural adaptations, and increase in strength and endurance [11].

Both have shown efficacy, but since they are different mechanisms of action, the time of effects and their evolution could be different.

The aim of our study is to compare two scientifically approved therapies for the NCNP in different stages of follow-up, one of them with a greater influence on neurophysiological effects as manual therapy and the other through therapeutic exercise involving reorganization in motor patterns and structural adaptations.

## Methods/design

### Trial design

The trial design is a randomized, controlled, parallel, double-blind, three-arm clinical trial of treatment.

### Hypothesis

Experimental treatments have a greater beneficial effect on disability and pain of subjects with NCNP than sham treatment.

### Sample selection

Individuals with NCNP were recruited through a text message broadcast on social networks in the city of Seville and were selected based on the eligibility criteria listed below. The study was carried out in facilities of the physiotherapy department of the University of Seville.

### Inclusion criteria

- Age 18–50 years
- Current neck pain
- Neck pain continued for at least the last 12 weeks [2].

### Exclusion criteria

- Irradiated neck pain
- Neck pain associated with vertigo
- Osteoporosis (Rx Control)
- Diagnosed psychological disorders
- Vertebral fractures (Rx Control)
- Tumors (Rx Control)
- Diagnosed metabolic diseases
- Previous neck surgery
- Red flags (night pain, severe muscle spasm, loss of involuntary weight, symptom mismatch)
- Physiotherapeutic treatment continued in the last 3 months

### Interventions

The participants could only receive the assigned treatment; they could not combine the treatment with drugs or other physiotherapeutic treatment. Any interference in the treatment was a reason for exclusion. The participants were warned that if they took any medication, they would be excluded; they were asked in all evaluations about the use of any type of medication.

### Group 1: Manual therapy

“Manual therapy” protocol was composed of three techniques based on scientific evidence for the treatment of neck pain [12–15].

This protocol was applied in the three treatment sessions, one per week.

1. High thoracic manipulation on T4. Patients are positioned supine with their arms crossed in a “V” shape over the chest. The therapist makes contact with the fist at the level of the spinous process of T4 and blocks the patient’s elbows with his chest. Following this, he introduces flexion of the cervical spine until a slight tension is felt in the tissues at the point of contact. Downward and cranial manipulation is applied. If cavitation is not achieved on the first attempt, the therapist repositions the patient and performs a second manipulation. A maximum of two attempts will be allowed in each patient [12].
2. Cervical articular mobilization (2 Hz, 2 min × 3 series). The patient is placed on the stretcher in a prone position, placing both hands under his forehead. The therapist makes contact with his two thumbs on the spinous process of the patient’s C2 vertebra and performs grade III posteroanterior impulses at a speed of 2 Hz and for 2 min. There are 3 mobilization intervals with a minute of rest between each one of them [13].
3. Suboccipital muscle inhibition (3 min). With the patient lying supine, the therapist places both hands under the subject’s head, by contacting their fingers on the lower edge of the occipital bone, and exerts constant and painless pressure in the anterior and cranial direction for 3 min [14, 15].

### Group 2: Therapeutic exercise

“Therapeutic exercise” protocol: this protocol is based on a progression in load composed of different phases: at first, activation and recruitment of deep cervical flexors [16]; secondly, isometric exercise deep and superficial flexors co-contraction [16], and finally, eccentric recruitment of flexors and extensors [16–18]. This protocol, as far as we know, has not been studied, but activation of this musculature during similar tasks to those of our protocol has been observed [16–18]. This protocol was taught to patients in the first session and was performed once a day during the 3 weeks of treatment, 21 sessions in total. It was reinforced by the physiotherapist in each of the three individual sessions.

Week 1: Exercises 1 and 2.

1. Cranio-cervical flexion (CCF) in a supine position with a towel in the posterior area of the neck (3 sets, 10 repetitions, 10 s of contraction each repetition with 10 s of rest).
2. CCF sitting (3 sets, 10 repetitions, 10 s of contraction each repetition with 10 s of rest)

Week 2: Exercises 1, 2, 3, and 4.

3. Co-contraction of deep and superficial neck flexors in supine decubitus (10 repetitions, 10 s of contraction with 10 s of rest).
4. Co-contraction of flexors, rotators, and lateral flexors. The patients performed cranio-cervical flexion, while the physiotherapist asked him/her to tilt, rotate, and look towards the same side while he/she opposes a resistance with his/her hand (10 repetitions, 10 s of contraction with 10 s of rest).

Week 3: Exercises 1, 2, 3, 4, 5, and 6.

5. Eccentric for extensors. With the patient seated, he/she should perform cervical extension. Then, he/she must realize a CCF and finish doing a cervical flexion (10 repetitions).
6. Eccentric for flexors. The patients, placed in a quadrupedal and neutral neck position, should perform neck flexion; then, they must have done a cranio-cervical flexion and, maintaining that posture, extend the neck and then finally lose the CCF (10 repetitions).

### Group 3: Sham treatment

For the “control” protocol, the patients were placed in the supine position, while the physiotherapist placed his hands without therapeutic intention on the patient’s neck for 3 min. The physiotherapist simulated the technique of suboccipital inhibition [14]. Later, with the laser pointer off, patients were contacted without exerting pressure for 10 s. Patients assigned to the control group received treatment 1 or 2 after completing the study.

### Outcomes measures

As dependent variables of the study, we took pain, based on the visual analog scale (VAS) and the pressure pain threshold (PPT), and cervical disability, through the Neck Disability Index. VAS is a representative element of the perception of pain part of the subject; however, PPT focuses on a more objective part of pain; the combination of both gives us a great approximation to the actual measurement of pain, this being one of the elements more complex to reflect. The Neck Disability Index (NDI) allows us to obtain a very complete percentage of functionality/disability, since it includes multiple activities of daily life in its index.

- Neck Disability Index. The NDI is a self-assessment instrument of the specific functional status of subjects with neck pain with 10 elements that include pain, personal care, weight gain, reading, headache, concentration, work, driving, sleeping, and leisure. Each section is rated on a scale of 0 to 5, where 0 means “painless” and 5 means “the worst pain imaginable.” The points obtained are added to a total score. The questionnaire was interpreted as a percentage. The disability categories for NDI are 0–8%, without disability; 10–28%, mild; 30–48%, moderate; 50–64%, serious; and 70–100%, complete [19, 20].
- Visual analog scale for pain. The subjects participating in the study indicated the intensity of their pain by means of the VAS of 100 mm; they had to signal in a horizontal line of 100 mm where they would place their pain, being 0 mm “no pain” and the 100 mm “the worst pain imaginable” [21].
- Pressure pain threshold. The PPT was recorded in Newton/cm^2^ using digital algometer (Force Ten™ -Model FDX, Wagner, Greenwich, USA) with a surface area of round tip of 1 cm^2^. The measurement was taken on the spinous process of vertebra C2, the evaluator gradually increasing the pressure until the patient indicated through a “yes” when the pain or discomfort appeared. Three measurements were made, obtaining an average value of these three measurements for the statistical analysis [22, 23].

These variables were measured in the pre-evaluation, first evaluation (week 2), second evaluation (week 4, short-term), and third evaluation (week 12, medium term). These evaluations were carried out by an evaluator trained in these procedures; the data was stored in an Excel document.

### Sample size calculation

The sample size was calculated using the Granmo calculator v.7.12, based on the analysis of the variance of means, and estimating an alpha risk of 5% (0.05), a beta risk of 10% (0.10), in a unilateral contrast, a typical deviation of 12% (0.12), a minimum difference to detect of 13.5% (0.135) which is based as the minimum clinically important differences in VAS [24], and a rate of follow-up losses of 8%, for which 20 subjects are required in each group, assuming that there are three groups. Finally, we included 69 patients who were divided into three groups, each group of at least 20 subjects, being able to overcome this value to assume the possible loss of follow-up.

### Randomization

Subjects were divided into three groups by means of balanced randomization, performed with free software (http://www.randomized.org/). The randomization sequence was only known by the principal investigator and auditor.

### Blinding

Evaluator and participants in the study were blinded during the entire process. The evaluators were unaware of the study objectives and the allocation of patients in the study groups. The patients did not know the group to which they belonged. The sequence of randomization was hidden from the evaluators and the patients, and this sequence was kept by the main researcher throughout the study and was not available to the rest of the participants involved in the trial.

### Statistical analysis

The statistical analysis was carried out through the IBM-SPSS Statistics 24 software. The normality test applied to all the variables was Kolmogorov-Smirnov test. For the contrast of intragroup hypotheses, Student’s *T* test for paired variables was applied in the case of parametric distributions and Kruskal-Wallis *H* for non-parametric distributions. For the intergroup hypothesis contrast, one factor ANOVA will be used in the case of parametric distributions and Kruskal-Wallis *H* for nonparametric distributions. Post-hoc analysis was obtained through Bonferroni’s contrast for parametric distributions and Mann-Whitney’s *U* for nonparametric ones. The confidence level used will be 95% (0.05) and the power of the study will be 90% (0.1). Size effect was calculated through eta squared, where the values of *r*^2^ have been considered as 0.01 (small), 0.06 (medium), and 0.14 (large). Principles of intention to treat were not applied.

## Results

**Fig. 1:**
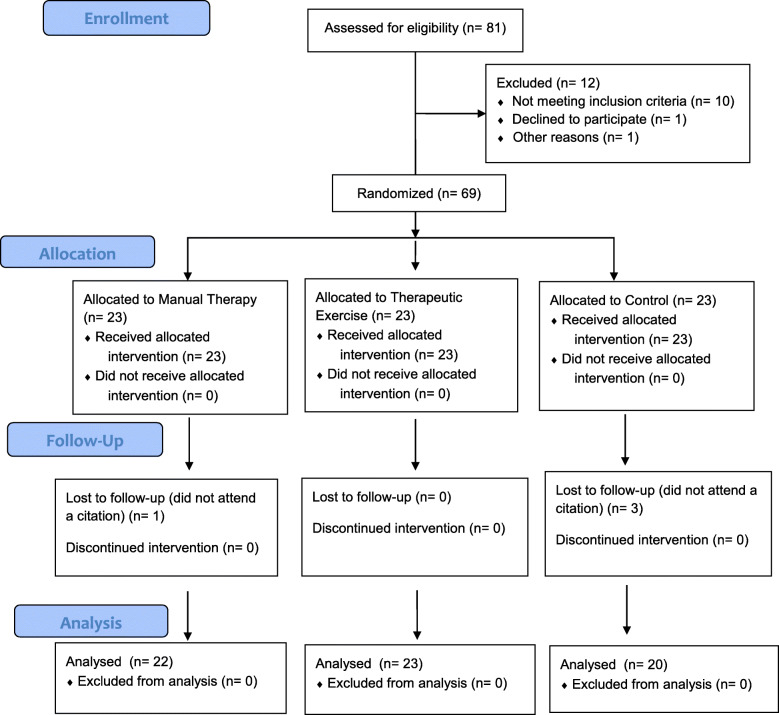
CONSORT flow diagram

After conducting 81 evaluations, 69 subjects started the study, which completed 65 the trial (94%). There were four losses of follow-up, one of them in manual therapy group (4% of the group) and three in control group (13% of the group). The CONSORT flow diagram can be seen in Fig. 1.

Participants’ baseline characteristics are presented in Table 1. There were not significant differences between groups previous at interventions.

**Table 1:** Initial characteristic of the subjects according to the study group

Variables	Group			*Z*
	*Manual therapy*	*Therapeutic exercise*	*Control*	*P*
**Age** (i)	42.95 ± 2.89	36.78 ± 2.89	36.90 ± 2.89	0.312^b^
**Gender** (ii) (male, female; %)	23 (5/22); 77 (17/22)	22 (5/23); 78 (18/23)	25 (5/20); 75 (15/20)	0.315^c^
**Height** (i) (cm)	168.05 ± 3.47	165.48 ± 2.10	168.85 ± 2.19	0.413^b^
**Weight** (i) (Kg)	69.86 ± 3.47	65.26 ± 2.35	70.45 ± 2.15	0.467^b^
**Body mass index** (i) (Kg/m^2^)	24.67 ± 1.13	23.8 ± 0.72	24.75 ± 0.75	0.379^b^
**VAS** (i) (mm)	41.95 ± 4.03	48.17 ± 3.48	49.80 ± 3.53	0.237^b^
**NDI** (i) (%)	27.32 ± 1.88	27.96 ± 2.02	29.55 ± 3.35	0.999^b^
**PPT**(i) (*N*)	21.79 ± 1.42	20.33 ± 1.31	23.55 ± 1.30	0.292^a^

*Control* control group, *Manual therapy* manual therapy group, *Therapeutic exercise* therapeutic exercise group, *Z* Shapiro-Wilk normality test, *P* statistical significance

(i) Dates expressed as means ± standard deviation

(ii) Dates expressed as percent (partial/total)

^a^ANOVA

^b^Kruskal-Wallis *H*

^c^Chi-squared

*Statistically significant differences between groups (*P* < 0.05)

Intragroup analysis shows significant improvements in VAS throughout the treatment process (weeks 1 and 4) and in the subsequent follow-up (week 12) in experimental treatments, manual therapy and therapeutic exercise. However, the control group did not show differences in VAS in any of the evaluations performed. For NDI, we observed that statistically significant differences were obtained in the first and second evaluation (weeks 1 and 4) for both experimental groups, and there were no changes in the control group. In the medium term, the MT group maintains the values obtained at the end of the treatment; however, the therapeutic exercise group shows a reduction in its results with respect to the second evaluation, although it continues to obtain significant results with respect to baseline. For PPT, the values obtained do not reflect changes in the first week of treatment for any of the three groups. After completing the complete treatment (second evaluation), we obtained statistically significant results only for the MT group, since the therapeutic exercise group shows an increase in PPT, but it is not enough to achieve statistical significance. After the follow-up (week 12), both experimental groups obtain statistically significant improvements and improve the resulting values in the second evaluation. The control group does not obtain improvements in any of the evaluations. The mean values and their statistical significance are shown in Table 2.

**Table 2:** Intragroup analysis. Changes to immediate, short, and medium terms according to study group

	VAS pre (i)	VAS week 1 (i)	VAS week 4 (i)	VAS week 12 (i)	NDI pre (i)	NDI week 1 (i)	NDI week 4 (i)	NDI week 12 (i)	PPT pre (i)	PPT week 1 (i)	PPT week 4 (i)	PPT week 12 (i)
**Manual therapy**	41.95 ± 4.03	32.77 ± 3.14	15.82 ± 3.26	18.23 ± 4.33	27.32 ± 8.80	22.00 ± 8.54	11.82 ± 2.00	11.23 ± 8.42	21.78 ± 1.42	21.51 ± 1.25	26.91 ± 1.41	26.79 ± 2.09
***P***		0.007*^b^	0.001*^b^	0.002*^b^		0.001^a^	0.001^b^	0.001^a^		0.806^a^	0.001^a^	0.003^a^
**Therapeutic exercise**	48.17 ± 3.48	35.83 ± 3.88	17.83 ± 3.42	24.43 ± 4.70	27.96 ± 9.68	19.17 ± 8.90	9.87 ± 1.78	13.09 ± 11.94	20.33 ± 1.31	20.70 ± 1.59	22.30 ± 1.84	25.22 ± 1.73
***P***		0.008*^a^	0.001*^b^	0.002*^b^		0.001^a^	0.001^b^	0.001^b^		0.729^a^	0.061^a^	0.001^a^
**Control**	49.80 ± 3.53	49.25 ± 3.53	50.55 ± 3.54	48.75 ± 3.51	29.38 ± 14.63	31.14 ± 15.84	31.20 ± 3.51	29.90 ± 14.66	23.33 ± 1.26	23.12 ± 1.22	23.23 ± 1.17	23.18 ± 1.14
***P***		0.342^a^	0.518^a^	0.337^a^		0.194^b^	0.105^b^	0.505^b^		0.566^a^	0.673^a^	0.634^a^

*Control* control group, *Manual therapy* manual therapy group, *Therapeutic exercise* therapeutic exercise group, *P* statistical significance from tests, *VAS* visual analog scale (mm), *NDI* Neck Disability Index (%), *PPT* pressure pain threshold (*N*/cm^2^)

(i) Dates expressed as means ± standard deviation

^a^Student’s *t* for paired samples

^b^Wilcoxon Z

*Statistically significant differences between groups (*P* < 0.05)

Intergroup analysis shows that in the first evaluation (after 1 week of treatment), only significant differences are observed between groups in the NDI, where there are greater improvements in the therapeutic exercise group when compared to the manual therapy group. Both treatments achieve improvements when compared to control. For VAS and PPT, no group shows statistically significant differences in the first week. In the second evaluation (week 4), the experimental groups show significant differences with respect to the control in VAS and NDI, without appreciating differences between them. However, for PPT only, the MT group achieves superior results to the control. The statistical analysis does not show significant differences between the experimental groups, but its *P* value (0.072) borders on the statistical significance. In the last evaluation (week 12), the experimental groups show differences with respect to the control in all the variables, VAS, NDI, and PPT. They do not reflect differences between experimental treatments for any of the variables studied. The values of statistical significance between groups are shown in Table 3.

**Table 3:** Intergroup analysis. Statistical significance between groups

Variable	*P* Week 1	Post-hoc	*P*	*P* Week 4	Post-hoc	*P*	*P* Week 12	Post-hoc	*P*
**VAS**	0.067^a^	*MT*–*C*	0.329^c^	0.001*^a^	*MT*–*C*	0.001*^c^	0.003*^a^	*MT*–*C*	0.008*^c^
		*TE*–*C*	0.070^c^		*TE*–*C*	0.001*^c^		*TE*–*C*	0.007*^c^
		*MT*–*TE*	1.000^c^		*MT*–*TE*	1.000^c^		*MT*–*TE*	1.000^c^
**NDI**	0.001*^b^	*MT*–*C*	0.001*^d^	0.001*^b^	*MT*–*C*	0.001*^d^	0.001*^a^	*MT*–*C*	0.001*^c^
		*TE*–*C*	0.001*^d^		*TE*–*C*	0.001*^d^		*TE*–*C*	0.001*^c^
		*MT*–*TE*	0.031*^d^		*MT*–*TE*	0.356^d^		*MT*–*TE*	1.000^c^
**PPT**	0.853^b^	*MT*–*C*	0.743^d^	0.002*^a^	*MT*–*C*	0.001*^c^	0.001*^b^	*MT*–*C*	0.012*^d^
		*TE*–*C*	0.991^d^		*TE*–*C*	0.415^c^		*TE*–*C*	0.001*^d^
		*MT*–*TE*	0.532^d^		*MT*–*TE*	0.072^c^		*MT*–*TE*	0.674^d^

*C* control group, *MT* manual therapy group, *TE* therapeutic exercise group, *P* statistical significance

^a^ANOVA

^b^Kruskal-Wallis *H*

^c^Bonferroni

^d^Mann-Whitney *U*

*Statistically significant differences between groups (*P* < 0.05)

The analysis performed for the effect size shows for VAS medium sizes in the first week of treatment and large sizes after treatment and subsequent follow-up for both experimental groups. For NDI, the analysis reflects large sizes in all evaluations for both experimental groups (*r*^2^ < 0.14). For PPT, the size of the effect in the first week fails to demonstrate the effect since its value is very low; in the second evaluation (week 4), the MT group reflects a large and medium effect size for the TE group. In the third evaluation, both experimental groups obtain large effect sizes. The effect size values are shown in Table 4.

**Table 4:** Size effect. Clinical significance of the interventions with respect to the control group

	*R*^2^ Week 1	*R*^2^ Week 4	*R*^2^ Week 12
**Manual therapy VAS**	0.094	0.373	0.270
**Therapeutic exercise VAS**	0.113	0.496	0.209
**Manual therapy NDI**	0.283	0.543	0.569
**Therapeutic exercise NDI**	0.430	0.629	0.459
**Manual therapy PPT**	0.001	0.265	0.214
**Therapeutic exercise PPT**	0.006	0.082	0.328

*VAS* visual analog scale, *NDI* Neck Disability Index, *PPT* pressure pain threshold. *R*^2^ 0.01 (small), 0.06 (medium), 0.14 (large)

## Discussion

The objective of this study was to determine the differences between experimental treatments in immediate, short, and medium terms, in addition to checking its effectiveness through a control group. Other studies have analyzed the differences between manual therapy and therapeutic exercise. However, they have not included complete clinical performance protocols, but isolated manual therapy or exercise techniques [25]. We believe that our intervention protocols could be considered as treatment options by themselves, without adding other complementary techniques.

The results obtained by the two experimental treatments with respect to the control showed a clear efficacy of these in subjects with nonspecific chronic neck pain, which showed statistically significant improvements and very high effect sizes in the short and medium terms in disability and perceived pain.

Regarding the comparison between the experimental treatments, we found that disability, measured through NDI, showed statistically significant differences in the immediate effects in favor of the therapeutic exercise group. In the short and medium terms, there were no statistically significant differences between the experimental groups, with no high differences observed in their means. This suggests that therapeutic exercise is faster in obtaining a decrease in cervical disability, but both treatments achieve great improvements on disability.

For the assessment of perceived pain, we evaluated two variables: VAS and PPT. The experimental treatments did not obtain significant results with respect to the control group. This can be explained by the neurophysiological effects of the placebo that obtains improvements in immediate short term [26]. In the second (week 4) and third evaluations (week 12), statistically significant results were obtained with respect to the control and not among the experimental treatments in the short term and in the medium term. All of them are exceeding 15 mm and therefore clinically relevant [24]. For PPT, in immediate terms, no group obtained differences with respect to control; however, in the short term, the MT group does obtain differences with control, while TE did not obtain them. This could be explained by the neurophysiological effects of manual therapy [10], acting faster at the local level, since in the medium term both experimental groups differ with the control obtaining statistically significant means 12 weeks after the start of treatment.

Our results obtained overall improvements in disability and pain perceived following the two experimental treatments in the short and medium terms, manual therapy and therapeutic exercise for the neck. The latest systematic reviews rate the effect of these interventions as moderate [8, 9]. However, our study did not analyze long-term changes, this being an important limitation in subjects with chronic pain. In addition and as main limitations, our study does not have a large sample size, so a larger sample size could refute our findings with greater determination. No method has been followed that guarantees compliance with home exercises by patients assigned to the therapeutic exercise group; they were only asked if they had done it daily.

Analyzing the results obtained by our study, we found that no significant differences in the short and medium terms between two very different treatments, which act through different mechanisms of action, were obtained. We want to support the latest advances in pain neurophysiology, which begin to elucidate the mechanisms and changes produced in the central nervous system that are activated by any type of treatment. In addition, influenced by psychosocial and environmental factors, lifestyle, and physical activity [27–29], the effectiveness of a cognitive approach to pain refutes our approach; there are studies that support this argument [30]. Clinical improvement could potentially be influenced by central processes.

As clinical implications, we believe that, although the object of our study is a comparison between two treatments, the results of the study make us aware of the importance of the combination of therapies. We should not get over manual techniques since their neurophysiological effects produce a reduction of pain more quickly, but we must also indicate specific therapeutic exercises since they will generate an improvement in disability before. In addition, exercise therapy encourages active treatment that could help in reducing catastrophism and fear of movement so common in these patients [31].

A multimodal approach based on manual therapy, therapeutic exercise, and pain education could be the best therapeutic weapon for subjects with nonspecific chronic neck pain. Future studies should analyze the effects of multimodal treatment, analyzing the central sensitization component of these subjects with chronic pain and the sleep-rest component and carrying out long-term follow-up.

## Conclusion

Both experimental treatments, manual therapy and therapeutic exercise, produce statistically significant and clinically relevant changes with respect to the control group. There are no statistically significant differences between the experimental groups in the short and medium terms. The therapeutic exercise group reduces cervical disability before manual therapy group does. The manual therapy group reduces pain perception before than therapeutic exercise group does.

## Data Availability

The datasets used and/or analyzed during the current study are available from the corresponding author on reasonable request.

## Abbreviations

NCNP: Non-specific chronic neck pain
PS: Postural stability
CCF: Cranio-cervical flexion
NDI: Neck Disability Index
VAS: Visual analog scale
PPT: Pressure pain threshold
OBI: Overall Balance Index

## References

[1] Childs JD, Cleland JA, Elliott JM, Teyhen DS, Wainner RS, Whitman JM (2008). Neck pain: clinical practice guidelines linked to the International Classification of Functioning, Disability, and Health from the Orthopedic Section of the American Physical Therapy Association. J Orthop Sports Phys Ther.

[2] Cohen SP (2015). Epidemiology, diagnosis, and treatment of neck pain. Mayo Clin Proc England.

[3] Daffner SD, Hilibrand AS, Hanscom BS, Brislin BT, Vaccaro AR, Albert TJ (2003). Impact of neck and arm pain on overall health status. Spine (Phila Pa 1976).

[4] Safiri S, Kolahi AA, Hoy D, Buchbinder R, Mansournia MA, Bettampadi D, Ashrafi-Asgarabad A, Almasi-Hashiani A, Smith E, Sepidarkish M, Cross M, Qorbani M, Moradi-Lakeh M, Woolf AD, March L, Collins G, Ferreira ML (2020). Global, regional, and national burden of neck pain in the general population, 1990-2017: systematic analysis of the Global Burden of Disease Study 2017. BMJ (Clinical research ed).

[5] Treleaven J (2008). Sensorimotor disturbances in neck disorders affecting postural stability, head and eye movement control. Man Ther.

[6] Ortego G, Villafañe JH, Doménech-García V, Berjano P, Bertozzi L, Herrero P (2016). Is there a relationship between psychological stress or anxiety and chronic nonspecific neck-arm pain in adults? A systematic review and meta-analysis. J Psychosom Res.

[7] Cuenca-martínez F, Bartrina-rodríguez I, Suso-martí L, La R, Ferrer-peña R, Bartrina-rodríguez I (2018). Association between somatosensory, motor and psychological variables by levels of disability in patients with cervicogenic dizziness. Somatosens Mot Res.

[8] Hidalgo B, Hall T, Bossert J, Dugeny A, Cagnie B, Pitance L (2017). The efficacy of manual therapy and exercise for treating non-specific neck pain: a systematic review. J Back Musculoskelet Rehabil.

[9] Fredin K, Lorås H (2017). Manual therapy, exercise therapy or combined treatment in the management of adult neck pain – a systematic review and meta-analysis. Musculoskelet Sci Pract.

[10] Bishop MD, Torres-Cueco R, Gay CW, Lluch-Girbés E, Beneciuk JM, Bialosky JE (2015). What effect can manual therapy have on a patient’s pain experience?. Pain Manag.

[11] Jull GA, Falla D, Vicenzino B, Hodges PW (2009). The effect of therapeutic exercise on activation of the deep cervical flexor muscles in people with chronic neck pain. Man Ther.

[12] Saavedra-Hernandez M, Arroyo-Morales M, Cantarero-Villanueva I, Fernandez-Lao C, Castro-Sanchez AM, Puentedura EJ (2013). Short-term effects of spinal thrust joint manipulation in patients with chronic neck pain: a randomized clinical trial. Clin Rehabil England.

[13] Lopez-Lopez A, Alonso Perez JL, Gonzalez Gutierez JL, La Touche R, Lerma Lara S, Izquierdo H (2015). Mobilization versus manipulations versus sustain apophyseal natural glide techniques and interaction with psychological factors for patients with chronic neck pain: randomized controlled trial. Eur J Phys Rehabil Med.

[14] Jeong E-D, Kim C-Y, Kim S-M, Lee S-J, Kim H-D (2018). Short-term effects of the suboccipital muscle inhibition technique and cranio-cervical flexion exercise on hamstring flexibility, cranio-vertebral angle, and range of motion of the cervical spine in subjects with neck pain: a randomized controlled trial. J Back Musculoskelet Rehabil.

[15] Heredia-Rizo AM, Pascual-Vaca AO, Cabello MA (2012). Immediate effects of the suboccipital muscle inhibition technique in craniocervical posture and greater occipital nerve mechanosensitivity in subjects with a history of orthodontia use: a randomized trial. J Manip Physiol Ther.

[16] Gross AR, Paquin JP, Dupont G, Blanchette S, Lalonde P, Cristie T (2016). Exercises for mechanical neck disorders: a Cochrane review update. Man Ther.

[17] Schomacher J, Falla D (2013). Function and structure of the deep cervical extensor muscles in patients with neck pain. Man Ther.

[18] Elliot JM, O’Leary SP, Cagnie B, Durbridge G, Danneels L, Jull G (2010). Craniocervical orientation affects muscle activation when exercising the cervical extensors in healthy subjects. Arch Phys Med Rehabil.

[19] Cleland JA, Fritz JM, Whitman JM, Palmer JA (2006). The reliability and constructo validity of the neck disability index and patient specific functional scale in patients with cervical radiculopathy. Spine.

[20] Kovacs FM, Bagó J, Royuela A, et al. Psychometric characteristics of the Spanish version of instruments to measure neck pain disability. BMC Musculoskelet Disord. 2008;9:42. 10.1186/1471-2474-9-42. 10.1186/1471-2474-9-42 PMC2375887 18400084

[21] Price DD, McGrath PA, Rafii A, Buckingham B (1983). The validation of visual analogue scales as ratio scale measures for chronic and experimental pain. Pain.

[22] Fischer AA (1998). Algometry in diagnosis of musculoskeletal pain and evaluation of treatment outcome: an update. J Muscoskel Pain.

[23] Chesterton LS, Sim J, Wright CC, Foster NE (2007). Interrater reliability of algometry in measuring pressure pain thresholds in healthy humans, using multiple raters. Clin J Pain.

[24] Kovacs FM, Abraira V, Royuela A, Corcoll J, Alegre L, Tomas M, Cano A, Muriel A, Zamora J, DelReal MT, Gestoso M, Mufraggi N (2008). Minimum detectable and minimal clinically important changes for pain in patients with nonspecific neck pain. BMC Musculoskelet Disord.

[25] Lluch E, Schomacher J, Gizzi L, Petzke F, Seegar D, Falla D (2014). Immediate effects of active cranio-cervical flexion exercise versus passive mobilisation of the upper cervical spine on pain and performance on the cranio-cervical flexion test. Man Ther.

[26] Rossettini G, Carlino E, Testa M (2018). Clinical relevance of contextual factors as triggers of placebo and nocebo effects in musculoskeletal pain. BMC Musculoskelet Disord.

[27] Nijs J, Loggia ML, Polli A, Moens M, Huysmans E, Goudman L (2017). Sleep disturbances and severe stress as glial activators: key targets for treating central sensitization in chronic pain patients?. Expert Opin Ther Targets.

[28] Nijs J, Goubert D, Ickmans K (2016). Recognition and treatment of central sensitization in chronic pain patients: not limited to specialized care. J Orthop Sports Phys Ther.

[29] Lluch Girbes E, Meeus M, Baert I, Nijs J (2015). Balancing “hands-on” with “hands-off” physical therapy interventions for the treatment of central sensitization pain in osteoarthritis. Man Ther.

[30] Beltran-Alacreu H, Lopez-de-Uralde-Villanueva I, Fernandez-Carnero J, La Touche R (2015). Manual therapy, therapeutic patient education, and therapeutic exercise, an effective multimodal treatment of nonspecific chronic neck pain: a randomized controlled trial. Am J Phys Med Rehabil.

[31] Muñoz-García D, Gil-Martínez A, López-López A, Lopez-de-Uralde-Villanueva I, La Touche R, Fernández-Carnero J (2016). Chronic neck pain and cervico-craniofacial pain patients express similar levels of neck pain-related disability, pain catastrophizing, and cervical range of motion. Pain Res Treat.

